# pyPESTO: a modular and scalable tool for parameter estimation for dynamic models

**DOI:** 10.1093/bioinformatics/btad711

**Published:** 2023-11-23

**Authors:** Yannik Schälte, Fabian Fröhlich, Paul J Jost, Jakob Vanhoefer, Dilan Pathirana, Paul Stapor, Polina Lakrisenko, Dantong Wang, Elba Raimúndez, Simon Merkt, Leonard Schmiester, Philipp Städter, Stephan Grein, Erika Dudkin, Domagoj Doresic, Daniel Weindl, Jan Hasenauer

**Affiliations:** Life and Medical Sciences (LIMES) Institute, University of Bonn, 53113 Bonn, Germany; Computational Health Center, Helmholtz Zentrum München Deutsches Forschungszentrum für Gesundheit und Umwelt (GmbH), 85764 Neuherberg, Germany; Department of Mathematics, Technical University of Munich, 85748 Garching, Germany; Department of Systems Biology, Harvard Medical School, Boston, MA 02115, United States; Life and Medical Sciences (LIMES) Institute, University of Bonn, 53113 Bonn, Germany; Life and Medical Sciences (LIMES) Institute, University of Bonn, 53113 Bonn, Germany; Life and Medical Sciences (LIMES) Institute, University of Bonn, 53113 Bonn, Germany; Computational Health Center, Helmholtz Zentrum München Deutsches Forschungszentrum für Gesundheit und Umwelt (GmbH), 85764 Neuherberg, Germany; Department of Mathematics, Technical University of Munich, 85748 Garching, Germany; Computational Health Center, Helmholtz Zentrum München Deutsches Forschungszentrum für Gesundheit und Umwelt (GmbH), 85764 Neuherberg, Germany; School of Life Sciences, Technical University of Munich, 85354 Freising, Germany; Computational Health Center, Helmholtz Zentrum München Deutsches Forschungszentrum für Gesundheit und Umwelt (GmbH), 85764 Neuherberg, Germany; Department of Mathematics, Technical University of Munich, 85748 Garching, Germany; Life and Medical Sciences (LIMES) Institute, University of Bonn, 53113 Bonn, Germany; Computational Health Center, Helmholtz Zentrum München Deutsches Forschungszentrum für Gesundheit und Umwelt (GmbH), 85764 Neuherberg, Germany; Department of Mathematics, Technical University of Munich, 85748 Garching, Germany; Life and Medical Sciences (LIMES) Institute, University of Bonn, 53113 Bonn, Germany; Computational Health Center, Helmholtz Zentrum München Deutsches Forschungszentrum für Gesundheit und Umwelt (GmbH), 85764 Neuherberg, Germany; Department of Mathematics, Technical University of Munich, 85748 Garching, Germany; Computational Health Center, Helmholtz Zentrum München Deutsches Forschungszentrum für Gesundheit und Umwelt (GmbH), 85764 Neuherberg, Germany; Department of Mathematics, Technical University of Munich, 85748 Garching, Germany; Leibniz Institute for Natural Product Research and Infection Biology, 07745 Jena, Germany; Life and Medical Sciences (LIMES) Institute, University of Bonn, 53113 Bonn, Germany; Life and Medical Sciences (LIMES) Institute, University of Bonn, 53113 Bonn, Germany; Life and Medical Sciences (LIMES) Institute, University of Bonn, 53113 Bonn, Germany; Computational Health Center, Helmholtz Zentrum München Deutsches Forschungszentrum für Gesundheit und Umwelt (GmbH), 85764 Neuherberg, Germany; Life and Medical Sciences (LIMES) Institute, University of Bonn, 53113 Bonn, Germany; Computational Health Center, Helmholtz Zentrum München Deutsches Forschungszentrum für Gesundheit und Umwelt (GmbH), 85764 Neuherberg, Germany; Department of Mathematics, Technical University of Munich, 85748 Garching, Germany

## Abstract

**Summary:**

Mechanistic models are important tools to describe and understand biological processes. However, they typically rely on unknown parameters, the estimation of which can be challenging for large and complex systems. pyPESTO is a modular framework for systematic parameter estimation, with scalable algorithms for optimization and uncertainty quantification. While tailored to ordinary differential equation problems, pyPESTO is broadly applicable to black-box parameter estimation problems. Besides own implementations, it provides a unified interface to various popular simulation and inference methods.

**Availability and implementation:**

pyPESTO is implemented in Python, open-source under a 3-Clause BSD license. Code and documentation are available on GitHub (https://github.com/icb-dcm/pypesto).

## 1 Introduction

**Figure 1.**
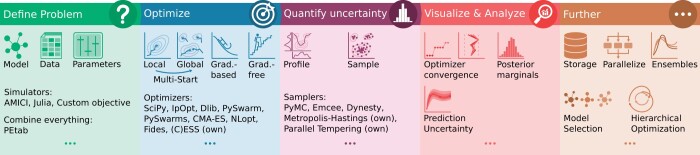
*Concept figure*. pyPESTO covers the full parameter estimation workflow, from problem definition (i.e. defining the parameter estimation problem based on dynamic model, data, parameters, and objective function), parameter optimization (i.e. finding parameters optimally explaining the data under the assumed objective function), uncertainty quantification (i.e. assessing uncertainty in parameter values), to visualization and analysis of results, and provides various features such as storage, parallelization, and advanced algorithms. See the main text for a contextualization of the key words indicated in the figure.

In many research areas, including computational biology, mathematical models are important tools to study complex systems and understand underlying mechanisms (Kitano 2002). While there are a variety of formalisms to describe biological systems, ordinary differential equation (ODE) models are popular as they provide natural means to describe and explain dynamic changes after perturbations, common in experimental biology (Fröhlich *et al.* 2019). However, models usually have unknown parameters that need to be estimated—their value and uncertainty—from observed data (Tarantola 2005). We present pyPESTO, a Python-based parameter estimation tool that provides various inference approaches in a modular manner via a streamlined pipeline (Fig. 1; see the Supplementary Information, Section 1, for a tool comparison). pyPESTO originated as a freely accessible reimplementation of the MATLAB tool PESTO (Stapor *et al.* 2018), but now offers a pipeline with a much broader spectrum of modern methods and interfaced tools. See the Supplementary Information, Section 2, for an example workflow including problem definition, optimization and uncertainty quantification, demonstrating the usefulness of a streamlined pipeline and unified interface. pyPESTO can be applied to various problem types, including large-scale problems.

## 2 Features

### 2.1 Problem definition

To ease application to biological systems, pyPESTO supports the community standard PEtab. PEtab is an open-source interoperable format for the specification of parameter estimation problems in systems biology, covering model definition, data, parameters, and noise model (Schmiester *et al.* 2021). For simulation, pyPESTO interfaces in particular the ODE simulation and sensitivity engine AMICI (Fröhlich *et al.* 2021) and provides an interface to objective functions written in the Julia language, in particular DifferentialEquations.jl (Rackauckas and Nie 2017). Furthermore, pyPESTO allows for user-defined continuous parameter estimation problems given via scalar objective functions or vector-valued residual functions. This includes log-likelihood and log-posterior based objective functions, where estimated values and uncertainties can be interpreted statistically. As many inference methods benefit from objective function derivatives, pyPESTO supports user-supplied functions that compute derivatives. In particular, in combination with AMICI, gradients via forward and adjoint sensitivity analysis, which is substantially more scalable for large-scale problems, are available (Fröhlich *et al.* 2021). Further, pyPESTO provides central, backward and forward finite differences with adaptive step size selection.

### 2.2 Optimization

Finding globally optimal parameters as point estimates is a common starting point in parameter estimation. As many problems are nonconvex and possess multiple local optima, globalization strategies are necessary. To this end, pyPESTO provides interfaces to global optimizers as well as a multi-start globalization strategy for local and global optimizers, which performs well for biological problems (Raue *et al.* 2013). pyPESTO provides a unified interface to local and global optimization libraries such as Ipopt (Wächter and Biegler 2006), Dlib (King 2009), PySwarms (Miranda 2018), pycma (Hansen 2022), SciPy (Virtanen *et al.* 2020), NLopt (Johnson and Schueller 2021), and Fides (Fröhlich and Sorger 2022). An extension to other tools is easily possible. Moreover, pyPESTO provides a hierarchical approach to efficiently handle relative data and noise parameters (Schmiester *et al.* 2019) and ordinal data (Schmiester *et al.* 2020). Hierarchical optimization essentially decomposes the optimization problem into dynamic parameters (e.g. reaction rates) and static parameters (e.g. scaling factors and noise variables) that do not affect the dynamics but only the observation function. This decomposition results in an optimization problem that can be substantially easier to solve.

### 2.3 Uncertainty analysis

For uncertainty analysis, pyPESTO implements the optimization based (frequentist) profile likelihood and (Bayesian) profile posterior approaches using the aforementioned interfaces to different optimization libraries (Raue *et al.* 2009). Moreover, pyPESTO provides Bayesian sampling algorithms. It implements a basic Metropolis Markov-chain Monte Carlo algorithm with adaptive estimation of the correlation structure and acceptance rate based scaling (Miasojedow *et al.* 2013, Łącki and Miasojedow 2015, Ballnus *et al.* 2017), and a modular parallel tempering framework based on Vousden *et al.* (2016). Parallel tempering allows to traverse the posterior landscape at different “temperatures” and is highly suited for multi-modal problems. Further, pyPESTO provides a unified interface to the popular sampling tools Emcee (Foreman-Mackey *et al.* 2013), PyMC (Salvatier *et al.* 2016) with custom gradients, and Dynesty (Speagle 2020).

### 2.4 Further aspects

pyPESTO provides various routines to visualize and analyze obtained results. Results can be saved, recovered, and shared in a compact storage format based on HDF5. Moreover, pyPESTO supports shared-memory parallelization via multi-threading and multi-processing. Thus, pyPESTO can be deployed flexibly on desktop machines as well as high-performance-computing infrastructure.

### 2.5 Limitations

While broadly applicable and providing a streamlined pipeline for parameter estimation implementing and interfacing many inference algorithms, pyPESTO clearly also has some limitations. First, it focuses on parameter inference; for model simulation, it relies on tools such as AMICI and DifferentialEquations.jl. While any objective function and simulator can be used, some functionality of pyPESTO, such as model-data fit visualization and built-in PEtab support, are currently easiest to use in combination with ODE models simulated via AMICI. AMICI itself is a highly efficient C++-based ODE simulation and sensitivity calculation engine that supports a broad spectrum of ODE problems, including e.g. pre-equilibration, discrete events, parameterized likelihoods and different noise models; for details see Fröhlich *et al.* (2021). Further, while pyPESTO makes a wide range of inference methods and tools accessible, there are also some that it does not interface yet (e.g. currently Stan and JAX). Due to its modular interface, adding more tools is however straightforward when there is demand. Moreover, presently pyPESTO does not directly provide local or global sensitivity or identifiability methods, besides the provided uncertainty analysis methods. Last, in contrast to tools such as Copasi (Hoops *et al.* 2006), pyPESTO does not provide a graphical user interface (GUI) but is Python script-based, easiest to use e.g. with Jupyter notebooks.

## 3 Implementation and availability

pyPESTO is implemented in Python, open-source under a 3-Clause BSD license. The code, designed to be modular and extensible, is hosted on GitHub and can be installed from PyPI. Extensive documentation is hosted on ReadTheDocs, including numerous notebooks containing tutorials and outlining pyPESTO’s functionality. “Getting started” tutorials allow to quickly get familiar with the basics of pyPESTO and a typical workflow including (custom) problem formulation, optimization, uncertainty quantification, visualization and analysis, and storage. Notebooks on “PEtab and AMICI” allow to quickly get familiar with workflows based on these tools. A further section on “Algorithms and features” provides detailed notebooks covering most of pyPESTO’s components in depth. The “API documentation” provides a detailed description of the implemented functionality, including links to the code and to method details for further information. We ensure correctness during development via unit tests and continuous integration. pyPESTO is primarily targeted at Linux, which is of particular interest for high-performance computations, and is also being continually tested on MacOS and Windows.

## 4 Discussion

pyPESTO has already been used in several research projects and publications, and is continuously being developed as a community tool by core contributors from different institutions. In the future, we plan to implement additional optimization and uncertainty quantification algorithms, to interface pyPESTO with further popular tools, and to extend and further standardize the supported parameter estimation workflows. We anticipate that pyPESTO will continue to be useful in a variety of computational biology applications and beyond.

## Supplementary Material

btad711_Supplementary_Data
