# Supplementary material for: pyPESTO: a modular and scalable tool for parameter estimation for dynamic models
Source: Bioinformatics. 2023 Nov 23;39(11):btad711. doi: 10.1093/bioinformatics/btad711 (PMC10689677; doi:10.1093/bioinformatics/btad711)
Supplement: btad711_Supplementary_Data [file btad711_supplementary_data.pdf]

Supplementary Information to:

pyPESTO: A modular and scalable tool for parameter estimation for dynamic models

Yannik Schälte[1,2,3,*], Fabian Fröhlich[4,*], Paul J. Jost[1,*], Jakob Vanhoefer[1,*], Dilan Pathirana[1], Paul Stapor[2,3], Polina Lakrisenko[2,5], Dantong Wang[2,3], Elba Raimúndez[1,2,3], Simon Merkt[1], Leonard Schmiester[2,3], Philipp Städter[2,3,6], Stephan Grein[1], Erika Dudkin[1], Domagoj Doresic[1], Daniel Weindl[2], and Jan Hasenauer[1,2,3,†]

[1]Life and Medical Sciences (LIMES) Institute, University of Bonn, Bonn 53113, Germany
[2]Computational Health Center, Helmholtz Zentrum München Deutsches Forschungszentrum für Gesundheit und Umwelt (GmbH), Neuherberg 85764, Germany
[3]Department of Mathematics, Technische Universität München, Garching 85748, Germany
[4]Department of Systems Biology, Harvard Medical School, Boston, MA 02115, USA
[5]School of Life Sciences, Technical University of Munich, Freising 85354, Germany
[6]Leibniz Institute for Natural Product Research and Infection Biology, Jena 07745, Germany,
[*]*These authors contributed equally*
[†]*Contact: jan.hasenauer@uni-bonn.de*

Contents

1 Comparison of simulation and inference tools features **2**

2 Coded example of a typical workflow **4**

1 Comparison of simulation and inference tools features

Table 1: Comparison of features of various systems biology simulation and inference tools.

Tool	Supported models	Modeling/data standards	Optimization				Uncertainty analysis				Parallelization
							Profiles		Sampling		
			Local	Global	Multi-start	Hierarchical	Global	Local approx.	MCMC	PT	
AMICI	ODE	SBML, PySB, BNGL, PEtab	N/A	N/A	N/A	N/A	N/A	N/A	N/A	N/A	OpenMP
AMIGO2	Custom	No	Yes	Yes	Yes	No	No	Yes	No	No	No
Copasi	ODE, SDE, MJP	SBML, PEtab	Yes	Yes	No	No	Yes	Yes	No	No	No
data2dynamics	ODE	PEtab	Yes	Yes	Yes	No	Yes	Yes	Yes	No	pthreads, parfor
dmod	ODE	PEtab	Yes	Yes	Yes	No	Yes	No	No	No	mclapply, dopar
MEIGO64	Custom	PEtab	Yes	Yes	Yes	No	No	No	Yes	No	jPar
parPE	ODE	PEtab	Yes	No	Yes	Yes	No	No	No	No	MPI
PEPSSBI	ODE	SBML	Yes	Yes	Yes	No	No	No	No	No	Various
PESTO	Custom; exp. ODE	No	Yes	Yes	Yes	Yes	Yes	Yes	Yes	Yes	parfor
pyBioNetFit	ODE	BNGL, SBML	Yes	Yes	No	No	No	No	Yes	Yes	Various
Tellurium+SBstoat	ODE, MJP	SBML	Yes	Yes	Yes	No	No	No	No	No	No
pyPESTO	Custom; esp. ODE	PEtab	Yes	Yes	Yes	Yes	Yes	Yes	Yes	Yes	Various

Tool	Gradients	Model simulation	Model development	GUI
AMICI	FW and AJ	Yes	No	No
AMIGO2	FD	Yes	No	No
Copasi	FD	Yes	Yes (SBML)	Yes
data2dynamics	FD and FW	Yes	Yes (own format)	No
dmod	FW	Yes	Yes (own format)	No
MEIGO64	Can be passed	N/A	No	No
parPE	FW and AJ	N/A (uses AMICI)	No	No
PEPSSBI	FD and FW	Yes	Yes (own format)	No
PESTO	FD, e.g. FW + AJ can be passed	N/A (uses AMICI)	No	No
pyBioNetFit	No	N/A (BioNetGen + libroadrunner interfaces)	Yes (BNGL)	No
Tellurium+SBstoat	FW	Yes	Yes (various)	No
pyPESTO	FD, e.g. FW+AJ can be passed	N/A, general interface to simulators, esp. AMICI	No	No

Tool	Language	Last update	Link	Reference
AMICI	Python, C++	2023	`https://amici.readthedocs.io/en/latest/`	Fröhlich et al. (2021)
AMIGO2	MATLAB	2021	`https://sites.google.com/site/amigo2toolbox/`	Balsa-Canto et al. (2016)
Copasi	C++	2023	`https://copasi.org/`	Hoops et al. (2006)
data2dynamics	MATLAB	2023	`https://github.com/Data2Dynamics/d2d`	Raue et al. (2015)
dmod	R	2022	`https://github.com/dkaschek/dMod`	Kaschek et al. (2019)
MEIGO64	MATLAB	2023	`https://github.com/gingproc-IIM-CSIC/MEIGO64`	Egea et al. (2014)
parPE	C++	2023	`https://github.com/ICB-DCM/parPE`	Schmiester et al. (2020)
PEPSSBI	Java	2020	`https://bitbucket.org/andreadega/systems-biology-compiler`	Degasperi, Fey, and Kholodenko (2017)
PESTO	MATLAB	2020	`https://github.com/ICB-DCM/PESTO`	Stapor et al. (2018)
pyBioNetFit	Python	2023	`https://bionetfit.nau.edu/`	Mitra et al. (2019)
Tellurium+SBstoat	Python	2023	`https://github.com/sys-bio/SBstoat`	Choi et al. (2018)
pyPESTO	Python	2023	`https://pypesto.readthedocs.io/en/latest/`	This paper

Color Coding

- Better
- Worse
- Not applicable

Abbreviations

N/A	Not applicable (e.g. because the tool just does simulation, or just inference, but not both)
MCMC	(Single-chain) Markov-Chain Monte-Carlo
PT	Parallel Tempering
ODE	Ordinary differential equation
MJP	Discrete-space Markov jump process
FD	Finite differences
FW	Forward sensitivity calculation
AJ	Adjoint sensitivity calculation
GUI	Graphical user interface

This overview was compiled in May 2023. The assessment is subjective and to the best of our knowledge.

pyPESTO originated as a freely accessible reimplementation of the MATLAB tool PESTO (Stapor et al., 2018), but now offers a streamlined pipeline with a much broader spectrum of modern methods and interfaced tools. pyPESTO is primarily designed to work with ODE-based problems – its AMICI and PEtab interfaces simplify workflows substantially. However, pyPESTO also accepts custom user-defined objective functions as input, which can be any simulation program that takes a parameter vector and returns a floating objective function value. In order for profile likelihood calculation and sampling to be meaningful, the passed objective function must permit interpretation as a negative log-likelihood, or together with priors, a negative log-posterior.

2 Coded example of a typical workflow

Please find below a typical workflow of using pyPESTO, including problem definition, optimization and uncertainty quantification, visualization, analysis and storage. Different steps are contrasted with an alternative workflow from scratch, demonstrating the added benefit of using pyPESTO.

Notebook on Workflow Comparison

September 1, 2023

pyPESTO vs no pyPESTO

The objectives of this notebook are twofold:

1. **General Workflow:** We walk step-by-step through a process to estimate parameters of dynamical models. By following this workflow, you will gain a clear understanding of the essential steps involved and how they contribute to the overall outcome.
2. **Benefits of pyPESTO:** Throughout the notebook, we highlight the key advantages of using pyPESTO in each step of the workflow, compared to "doing things manually". By leveraging its capabilities, you can significantly increase efficiency and effectiveness when solving your parameter optimization tasks.

This notebook is divided into several sections, each focusing on a specific aspect of the parameter estimation workflow. Here's an overview of what you find in each section:

Contents

1. **Objective Function:** We discuss the creation of an objective function that quantifies the goodness-of-fit between a model and observed data. We will demonstrate how pyPESTO simplifies this potentially cumbersome process and provides various options for objective function definition.
2. **Optimization:** We show how to find optimal model parameters. We illustrate the general workflow and how pyPESTO allows to flexibly use different optimizers and to analyze and interpret results.
3. **Profiling:** After a successful parameter optimization, we show how pyPESTO provides profile likelihood functionality to assess uncertainty and identifiability of selected parameters.
4. **Sampling:** In addition to profiles, we use MCMC to sample from the Bayesian posterior distribution. We show how pyPESTO facilitates the use of different sampling methods.
5. **Result Storage:** This section focuses on storing and organizing the results obtained from the parameter optimization workflow, which is necessary to keep results for later processing and sharing.

By the end of this notebook, you'll have gained valuable insights into the parameter estimation workflow for dynamical models. Moreover, you'll have a clear understanding of the benefits pyPESTO brings to each step of this workflow. This tutorial will equip you with the knowledge and tools necessary to streamline your parameter estimation tasks and obtain accurate and reliable results.

[1]:
```python
# imports
import logging
import os
import random
from pprint import pprint
import amici
import matplotlib as mpl
import matplotlib.pyplot as plt
import numpy as np
import petab
import scipy.optimize
from IPython.display import Markdown, display
import pypesto.optimize as optimize
import pypesto.petab
import pypesto.profile as profile
import pypesto.sample as sample
import pypesto.store as store
import pypesto.visualize as visualize
import pypesto.visualize.model_fit as model_fit

mpl.rcParams['figure.dpi'] = 100
mpl.rcParams['font.size'] = 18

# for reproducibility
random.seed(1912)
np.random.seed(1912)

# name of the model
model_name = "boehm_JProteomeRes2014"

# output directory
model_output_dir = "tmp/" + model_name
```

1. Create an objective function

As application problem, we consider the model by Böhm et al., JProteomRes 2014, which describes, trained on quantitative mass spectronomy data, the process of dimerization of phosphorylated STAT5A and STAT5B, important transductors of activation signals of cytokine receptors to the nucleus. The model is available via the PEtab benchmark collection. For simulation, we use AMICI, an efficient ODE simulation and sensitivity calculation routine. PEtab is a data format specification that standardises parameter estimation problems in systems biology..

Without pyPESTO

To fit an (ODE) model to data, the model needs to be implemented in a simulation program as a function mapping parameters to simulated data. Simulations must then be mapped to experimentally observed data via formulation of a single-value cost function (e.g. squared or absolute differences, corresponding to a normal or Laplace measurement noise model). Loading the model

via PEtab and AMICI already simplifies these steps substantially compared to encoding the model and the objective function manually:

[3]:
```
# PEtab problem loading
petab_yaml = f"./{model_name}/{model_name}.yaml"
petab_problem = petab.Problem.from_yaml(petab_yaml)
# AMICI model complilation
amici_model = amici.petab_import.import_petab_problem(
    petab_problem, force_compile=True
)
```

AMICI allows us to construct an objective function from the PEtab problem, already considering the noise distribution assumed for this model. We can also simulate the problem for a parameter with this simple setup.

[4]:
```
# Simulation with PEtab nominal parameter values
print("PEtab benchmark parameters")
pprint(amici.petab_objective.simulate_petab(petab_problem, amici_model))

# Simulation with specified parameter values
parameters = np.array([-1.5, -5.0, -2.2, -1.7, 5.0, 4.2, 0.5, 0.8, 0.5])
ids = list(amici_model.getParameterIds())
ids[6:] = ["sd_pSTAT5A_rel", "sd_pSTAT5B_rel", "sd_rSTAT5A_rel"]

print("Individualized parameters")
pprint(
    amici.petab_objective.simulate_petab(
        petab_problem,
        amici_model,
        problem_parameters={x_id: x_i for x_id, x_i in zip(ids, parameters)},
        scaled_parameters=True,
    )
)
```

```
PEtab benchmark parameters
{'edatas': [<Swig Object of type 'std::vector< amici::ExpData * >::value_type'
at 0x12c917d80
  condition 'model1_data1' starting at t=0.0 with custom parameter scales,
constants, parameters
  16x3 time-resolved datapoints
    (48/48 measurements & 0/48 sigmas set)
  10x0 event-resolved datapoints
    (0/0 measurements & 0/0 sigmas set)
>],
 'llh': -138.22199656856435,
 'rdatas': [<ReturnDataView(<amici.amici.ReturnData; proxy of <Swig Object of
type 'std::unique_ptr< amici::ReturnData >::pointer' at 0x12c917c60> >)>],
 'sllh': None}
Individualized parameters
```

```
{'edatas': [<Swig Object of type 'std::vector< amici::ExpData * >::value_type'
at 0x12c7dadf0
  condition 'model1_data1' starting at t=0.0 with custom parameter scales,
constants, parameters
  16x3 time-resolved datapoints
    (48/48 measurements & 0/48 sigmas set)
  10x0 event-resolved datapoints
    (0/0 measurements & 0/0 sigmas set)
>],
 'llh': -185.54291970899519,
 'rdatas': [<ReturnDataView(<amici.amici.ReturnData; proxy of <Swig Object of
type 'std::unique_ptr< amici::ReturnData >::pointer' at 0x12c917060> >)>],
 'sllh': None}
```

We see that to call the objective function, we need to supply the parameters in a dictionary format. This is not really suitable for parameter estimation, as e.g. optimization packages usually work with (numpy) arrays. Therefore we need to create some kind of parameter mapping.

[5]:
```python
class Objective:
    """
    A very basic implementation to an objective function for AMICI, that can␣
 ↪call the objective function just based on the parameters.
    """

    def __init__(self, petab_problem: petab.Problem, model: amici.Model):
        """Constructor for objective."""
        self.petab_problem = petab_problem
        self.model = model
        self.x_ids = list(self.model.getParameterIds())
        # nned to change the names for the last ones
        self.x_ids[6:] = ["sd_pSTAT5A_rel", "sd_pSTAT5B_rel", "sd_rSTAT5A_rel"]

    def x_dct(self, x: np.ndarray):
        """
        Turn array of parameters to dictionary usable for objective call.
        """
        return {x_id: x_i for x_id, x_i in zip(self.x_ids, x)}

    def __call__(self, x: np.ndarray):
        """Call the objective function"""
        return -amici.petab_objective.simulate_petab(
            petab_problem,
            amici_model,
            problem_parameters=self.x_dct(x),
            scaled_parameters=True,
        )["llh"]
```

```
# Test it out
obj = Objective(petab_problem, amici_model)
pprint(obj(parameters))
```

```
185.54291970899519
```

Summary We have constructed a very basic functioning objective function for this specific problem. AMICI and PEtab already simplify the workflow compared to coding the objective from scratch, however there are still some shortcomings. Some things that we have not yet considered and that would require additional code are:

- What if we have **multiple simulation conditions**? We would have to adjust the parameter mapping, to use the correct parameters and constants for each condition, and aggregate results.
- What if our system starts in a **steady state**? In this case we would have to preequilibrate, something that AMICI can do, but we would need to account for that additionally (and it would most likely also include an additional simulation condition).
- For later analysis we would like to be able to not only get the objective function but also the **residuals**, something that we can change in AMICI but we would have to account for this flexibility additionally.
- If we **fix a parameter** (i.e. keeping it constant while optimizing the remaining parameters), we would have to create a different parameter mapping (same for unfixing a parameter).
- What if we want to include **prior knowledge** of parameters?
- AMICI can also calculate **sensitivities** (`sllh` in the above output). During parameter estimation, the inference (optimization/sampling/...) algorithm typically calls the objective function many times both with and without sensitivities. Thus, we need to implement the ability to call e.g. function value, gradient and Hessian matrix (or an approximation thereof), as well as combinations of these for efficiency.

This is most likely not the complete list but can already can get yield quite substantial coding effort. While each problem can be tackled, it is a lot of code lines, and we would need to rewrite it each time if we want to change something (or invest even more work and make the design of the objective function flexible).

In short: **There is a need for a tool that can account for all these variables in the objective function formulation**.

With pyPESTO

All the above is easily addressed by using pyPESTO's objective function implementation. We support a multitude of objective functions (JAX, Aesara, AMICI, Julia, self-written). For PEtab models with AMICI, we take care of the parameter mapping, multiple simulation conditions (including preequilibration), changing between residuals and objective function, fixing parameters, and sensitivity calculation.

While there is a lot of possibility for individualization, in its most basic form creating an objective from a petab file accounting for all of the above boils down to just a few lines in pyPESTO:

[6]:
```
petab_yaml = f"./{model_name}/{model_name}.yaml"

petab_problem = petab.Problem.from_yaml(petab_yaml)
```

```
# import the petab problem and generate a pypesto problem
importer = pypesto.petab.PetabImporter(petab_problem)
problem = importer.create_problem()

# call the objective to get the objective function value and (additionally) the␣
↪gradient
problem.objective(parameters, sensi_orders=(0, 1))
```

[6]:
```
(185.5429188951038,
 array([ 4.96886729e+02,  1.50715517e-01,  4.44258325e+01,  7.14778242e+02,
        -5.19647592e-05, -1.66953531e+02, -1.50846999e+02, -6.86643591e+01,
        -1.59022641e+01]))
```

2. Optimization

After creating our objective function, we can now set up an optimization problem to find model parameters that best describe the data. For this we will need

- parameter bounds (to restrict the search area; these are usually based on domain knowledge)
- startpoints for the multistart local optimization (as dynamical system constrained optimization problems are in most cases non-convex)
- an optimizer

Without pyPESTO

[7]:
```
# bounds
ub = 5 * np.ones(len(parameters))
lb = -5 * np.ones(len(parameters))

# number of starts
n_starts = 25

# draw uniformly distributed parameters within these bounds
x_guesses = np.random.random((n_starts, len(lb))) * (ub - lb) + lb

# optimize
results = []
for x0 in x_guesses:
    results.append(
        scipy.optimize.minimize(
            obj,
            x0,
            bounds=zip(lb, ub),
            tol=1e-12,
            options={"maxfun": 500},
            method="L-BFGS-B",
```

```
        )
    )
pprint(results[0])
```

```
  message: STOP: TOTAL NO. of f AND g EVALUATIONS EXCEEDS LIMIT
  success: False
   status: 1
      fun: 483.97739572289964
        x: [-3.352e+00 -2.216e+00 -1.774e+00 -1.776e+00  6.234e-01
             3.661e+00  1.261e+00  6.150e-01  2.480e-01]
      nit: 18
      jac: [ 2.192e+02  3.002e+02  7.278e+02 -2.483e+02  3.634e+02
            -3.849e+01 -3.175e+01 -1.073e+03 -4.504e+02]
     nfev: 520
     njev: 52
 hess_inv: <9x9 LbfgsInvHessProduct with dtype=float64>
```

We might want to change the optimizer, like e.g. NLopt

[8]:
```python
import nlopt

opt = nlopt.opt(nlopt.LD_LBFGS, len(parameters))
opt.set_lower_bounds(lb)
opt.set_upper_bounds(ub)

def nlopt_objective(x, grad):
    """We need a wrapper function of the kind f(x,grad) for nlopt."""
    r = obj(x)
    return r

opt.set_min_objective(nlopt_objective)
results = []
for x0 in x_guesses:
    try:
        result = opt.optimize(x0)
    except (
        nlopt.RoundoffLimited,
        nlopt.ForcedStop,
        ValueError,
        RuntimeError,
        MemoryError,
    ) as e:
        result = None
    results.append(result)

pprint(results[0])
```

```
array([-4.72537584, -3.8267521 ,  0.17032373,  0.5309411 ,  1.82691101,
       -0.13738387, -2.19222726,  2.40671846, -2.17865902])
```

We can already see that the NLopt library takes different arguments and has a different result output than scipy. In order to be able to compare them, we need to modify the code again. We would at the very least like the end objective function value, our starting value and some kind of exit message.

[9]:
```
results = []
for x0 in x_guesses:
    try:
        result = opt.optimize(x0)
        msg = 'Finished Successfully.'
    except (
        nlopt.RoundoffLimited,
        nlopt.ForcedStop,
        ValueError,
        RuntimeError,
        MemoryError,
    ) as e:
        result = None
        msg = str(e)
    res_complete = {
        "x": result,
        "x0": x0,
        "fun": opt.last_optimum_value(),
        "message": msg,
        "exitflag": opt.last_optimize_result(),
    }
    results.append(res_complete)

pprint(results[0])
```

```
'exitflag': 1,
'fun': 1150653011.8393009,
'message': 'Finished Successfully.',
'x': array([-4.72537584, -3.8267521 ,  0.17032373,  0.5309411 ,  1.82691101,
     -0.13738387, -2.19222726,  2.40671846, -2.17865902]),
'x0': array([-4.72537584, -3.8267521 ,  0.17032373,  0.5309411 ,  1.82691101,
     -0.13738387, -2.19222726,  2.40671846, -2.17865902])
```

This is smoothly running and does not take too much code. But again, we did not consider quite a few things regarding this problem:

- The Optimizer internally uses **finite differences**, which is firstly inefficient and secondly inaccurate, especially for very stiff models. Constructing sensitivities and integrating them into the optimization can be quite tedious.
- There is no **tracking of the history**, we only get the end points. If we want to analyze this in more detail we need to implement this into the objective function.
- Many times, especcially for larger models, we might want to **change the optimizer** depending on the performance. For example, for some systems other optimizers might perform better, e.g. a global vs a local one. A detailed analysis on this would require some setup, and

each optimizer takes arguments in a different form.

- For bigger models and more starts, **parallelization** becomes a key component to ensure efficency.
- Especially when considering multiple optimizers, the lack of a **quasi standardised result format** becomes apparent und thus one would either have to write a proper result class or individualize all downstream analysis for each optimizer.

With pyPESTO

Using pyPESTO, all the above is easily possible. A `pypesto.engine.MultiProcessEngine` allows to use parallelization, and `optimize.ScipyOptimizer` specifies to use a scipy based optimizer. Alternatively, e.g. try `optimize.FidesOptimizer` or `optimize.NLoptOptimizer`, all with consistent calls and output formats. The results of the single optimizer runs are filled into a unified pyPESTO result object.

[10]:
```
results_pypesto = optimize.minimize(
    problem=problem,
    optimizer=optimize.ScipyOptimizer(),
    n_starts=n_starts,
    engine=pypesto.engine.MultiProcessEngine(),
)
# a summary of the results
display(Markdown(results_pypesto.summary()))
```

Optimization Result

- number of starts: 25
- best value: 138.2224842858494, id=2
- worst value: 254.03736660792256, id=6
- number of non-finite values: 0
- execution time summary:
  - Mean execution time: 4.508s
  - Maximum execution time: 10.909s, id=1
  - Minimum execution time: 0.390s, id=16
- summary of optimizer messages:

Count	Message
17	CONVERGENCE: REL_REDUCTION_OF_F_<=_FACTR*EPSMCH
8	ABNORMAL_TERMINATION_IN_LNSRCH

- best value found (approximately) 2 time(s)
- number of plateaus found: 5

A summary of the best run:

Optimizer Result

- optimizer used: <ScipyOptimizer method=L-BFGS-B options={'maxfun': 1000, 'disp': False}>
- message: ABNORMAL_TERMINATION_IN_LNSRCH
- number of evaluations: 141
- time taken to optimize: 6.583s
- startpoint: [-4.32816355 -4.74870318 -1.74009129 -1.26420992 3.53977881 0.54755365 2.64804722 1.62058431 1.57747828]
- endpoint: [-1.56907929 -5. -2.2098163 -1.78589671 3.55917603 4.19771074 0.58569077 0.81885971 0.49858833]
- final objective value: 138.2224842858494
- final gradient value: [-0.00783036 0.05534759 0.00129469 -0.00675505 -0.00121895 0.00394696 -0.00021472 0.00294705 0.00089969]

Beyond the optimization itself, pyPESTO naturally provides various analysis and visualization routines:

[11]:
```
_, axes = plt.subplots(ncols=2, figsize=(12, 6), constrained_layout=True)
visualize.waterfall(results_pypesto, ax=axes[0])
visualize.parameters(results_pypesto, ax=axes[1]);
```

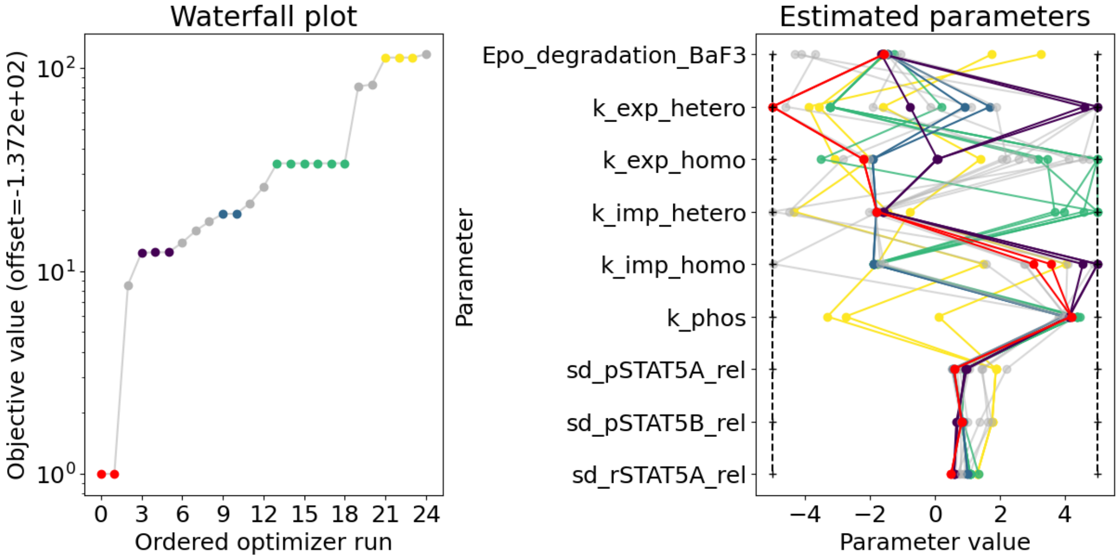

3. Profiling

Profile likelihood analysis allows to systematically asess parameter uncertainty and identifiability, tracing a maximum profile in the likelihood landscape starting from the optimal parameter value.

Without pyPESTO

When it comes to profiling, we have the main apparatus already prepared with a working optimizer and our objective function. We still need a wrapper around the objective function as well as the geneal setup for the profiling, which includes selecting startpoints and cutoffs. For the sake of computation time, we will limit the maximum number of steps the scipy optimizer takes to 50.

[16]:
```
# we optimimize the first parameter
x_start = results_pypesto.optimize_result[0]["x"][problem.x_free_indices][1:]
x_fixed = results_pypesto.optimize_result[0]["x"][problem.x_free_indices][0]
fval_min = results_pypesto.optimize_result[0]["fval"]

# determine stepsize, ratios
stepsize = 0.05
ratio_min = 0.145
x_profile = [results_pypesto.optimize_result[0]["x"][problem.x_free_indices]]
fval_profile = [results_pypesto.optimize_result[0]["fval"]]

# set up for nlopt optimizer
opt = nlopt.opt( nlopt.LD_LBFGS, len(parameters) - 1 )
opt.set_lower_bounds(lb[:-1])
opt.set_upper_bounds(ub[:-1])

for direction, bound in zip([-1, 1], (-5, 3)):  # profile in both directions
    x0_curr = x_fixed
    x_rest = x_start
    run = True
    while direction * (x0_curr - bound) < 0 and run:
        x0_curr += stepsize * direction

        # define objective for fixed parameter
        def fix_obj(x: np.ndarray):
            x = np.insert(x, 0, x0_curr)
            return obj(x)

        # define nlopt objective
        def nlopt_objective(x, grad):
            """We need a wrapper function of the kind f(x,grad) for nlopt."""
            r = fix_obj(x)
            return r

        opt.set_min_objective(nlopt_objective)
        result = opt.optimize(x_rest)

        # update profiles
        if direction == 1:
            x_profile.append(np.insert(result, 0, x0_curr))
            fval_profile.append(opt.last_optimum_value())
```

```
            if np.exp(fval_min - fval_profile[-1]) <= ratio_min:
                run = False
        if direction == -1:
            x_profile.insert(0, np.insert(result, 0, x0_curr))
            fval_profile.insert(0, opt.last_optimum_value())
            if np.exp(fval_min - fval_profile[0]) <= ratio_min:
                run = False
        x_rest = result
```

[17]:
```
plt.plot(
    [x[0] for x in x_profile], np.exp(np.min(fval_profile) - fval_profile)
);
```

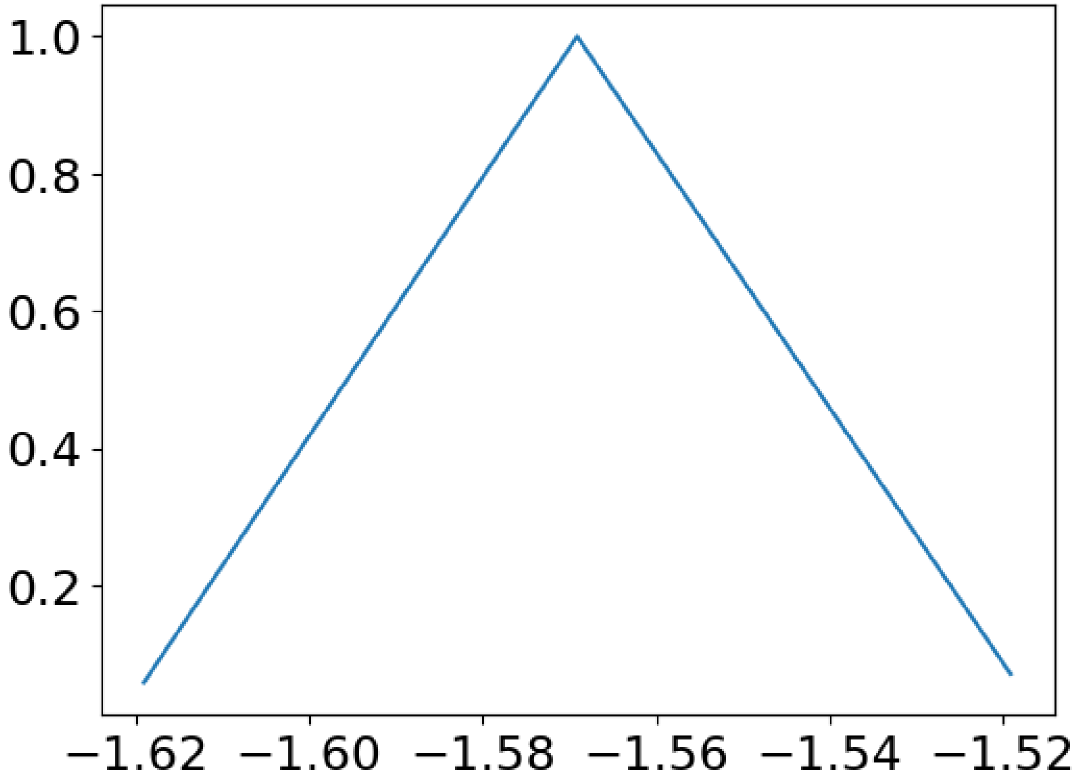

This is a very basic implementation that still lacks a few things:

- If we want to profile all parameters, we will want to **parallelize** this to save time.
- We chose a very unflexible stepsize, in general we would want to be able to automatically **adjust the stepsize** during each profile calculation.
- As this approach requires (multiple) optimizations under the hood, the things discussed in the last step also apply here mostly.

With pyPESTO

pyPESTO takes care of those things and integrates the profiling directly into the Result object

[18]:
```
result_pypesto = profile.parameter_profile(
    problem=problem,
    result=results_pypesto,
    optimizer=optimize.ScipyOptimizer(),
    engine=pypesto.engine.MultiProcessEngine(),
    profile_index=[0],
)

visualize.profiles(result_pypesto);
```

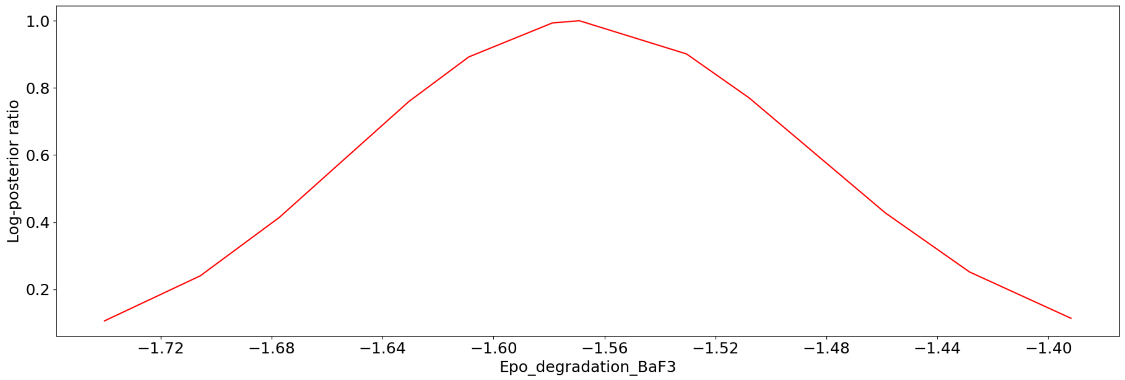

4. Sampling

pyPESTO also supports Bayesian sampling methods. These are used to retrieve posterior distributions and measure uncertainty globally.

Without pyPESTO

While there are many available sampling methods, setting them up for a more complex objective function can be time intensive, and comparing different ones even more so.

[19]:
```
import emcee

n_samples = 500


# set up the sampler
# rewrite nll to llh
def log_prob(x):
    """Log-probability density function."""
    # check if parameter lies within bounds
    if any(x < lb) or any(x > ub):
```

```
        return -np.inf
    # invert sign
    return -1.0 * obj(x)


# def a function to get multiple startpoints for walkers
def get_epsilon_ball_initial_state(
    center: np.ndarray,
    lb: np.ndarray,
    ub: np.ndarray,
    nwalkers: int = 20,
    epsilon: float = 1e-3,
):
    """Get walker initial positions as samples from an epsilon ball.

    The ball is scaled in each direction according to the magnitude of the
    center in that direction.

    It is assumed that, because vectors are generated near a good point,
    all generated vectors are evaluable, so evaluability is not checked.

    Points that are generated outside the problem bounds will get shifted
    to lie on the edge of the problem bounds.

    Parameters
    ----------
    center:
        The center of the epsilon ball. The dimension should match the full
        dimension of the pyPESTO problem. This will be returned as the
        first position.
    lb, ub:
        Upper and lower bounds of the objective.
    nwalkers:
        Number of emcee walkers.
    epsilon:
        The relative radius of the ball. e.g., if `epsilon=0.5`
        and the center of the first dimension is at 100, then the upper
        and lower bounds of the epsilon ball in the first dimension will
        be 150 and 50, respectively.
    """
    # Epsilon ball
    lb = center * (1 - epsilon)
    ub = center * (1 + epsilon)

    # Sample initial positions
    dim = lb.size
    lb = lb.reshape((1, -1))
```

```
    ub = ub.reshape((1, -1))

    # create uniform points in [0, 1]
    xs = np.random.random((nwalkers - 1, dim))

    # re-scale
    xs = xs * (ub - lb) + lb

    initial_state_after_first = xs

    # Include `center` in initial positions
    initial_state = np.row_stack(
        (
            center,
            initial_state_after_first,
        )
    )

    return initial_state


sampler = emcee.EnsembleSampler(
    nwalkers=18, ndim=len(ub), log_prob_fn=log_prob
)
sampler.run_mcmc(
    initial_state=get_epsilon_ball_initial_state(
        results_sorted[0]["x"], lb, ub, 18
    ),
    nsteps=n_samples,
    skip_initial_state_check=True,
)
```

[20]:
```
trace_x = np.array([sampler.get_chain(flat=True)])
trace_neglogpost = np.array([-sampler.get_log_prob(flat=True)])
```

[21]:
```
plt.plot(
    trace_neglogpost.reshape(
        9000,
    ),
    "o",
    alpha=0.05,
)
plt.ylim([240, 300]);
```

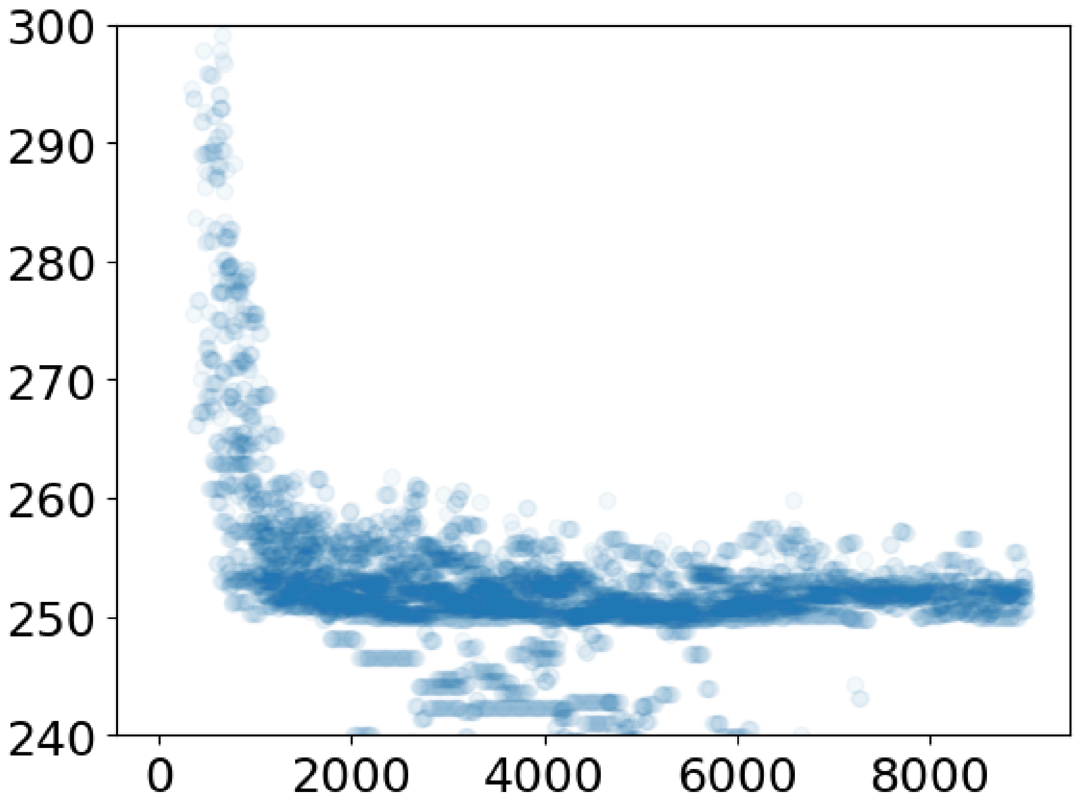

With pyPESTO

pyPESTO supports a number of samplers and unifies their usage, making a change of sampler comparatively easy. Instead of the below `sample.AdaptiveMetropolisSampler`, try e.g. also a `sample.EmceeSampler` (like above) or a `sample.AdaptiveParallelTemperingSampler`. It also unifies the result object to a certain extent to allow visualizations across samplers.

[22]:
```
# Sampling
sampler = sample.AdaptiveMetropolisSampler()
result_pypesto = sample.sample(
    problem=problem,
    sampler=sampler,
    n_samples=1000,
    result=result_pypesto,
)
```

```
100%|
| 1000/1000 [00:03<00:00,
311.04it/s]
Elapsed time: 3.9380469999998695
```

[23]:

```
# plot objective function trace
visualize.sampling_fval_traces(result_pypesto);
```

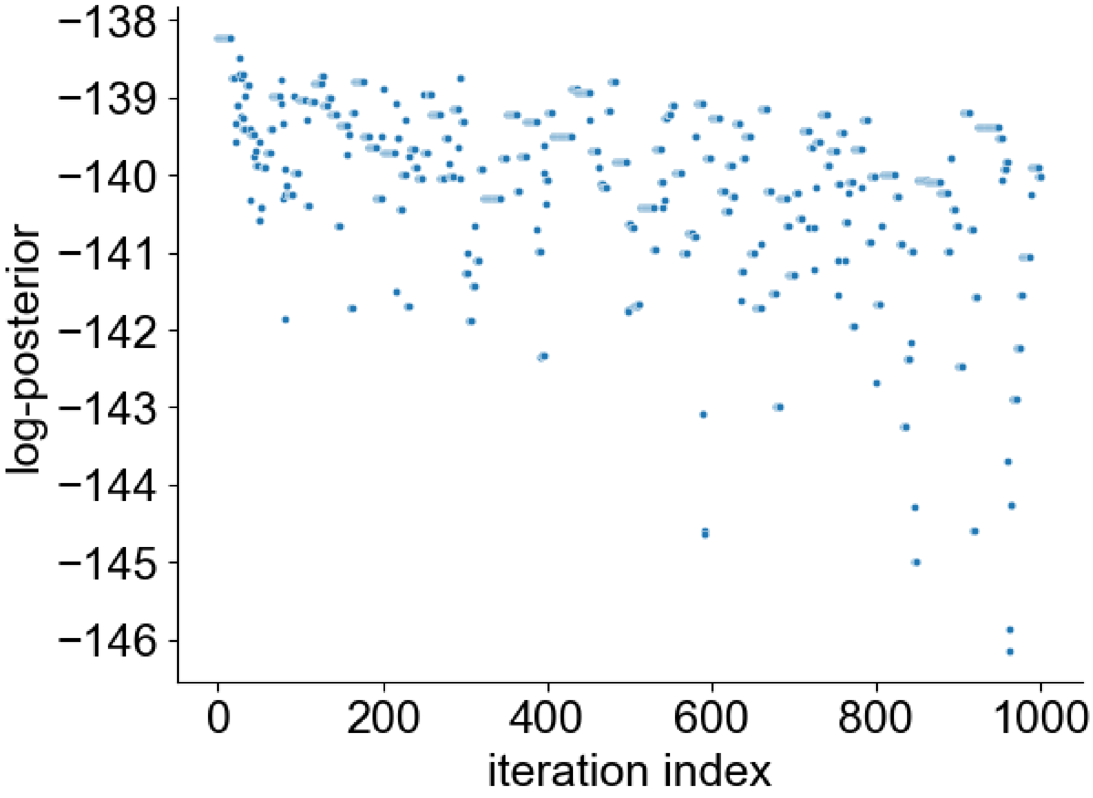

[24]:

```
visualize.sampling_1d_marginals(result_pypesto);
```

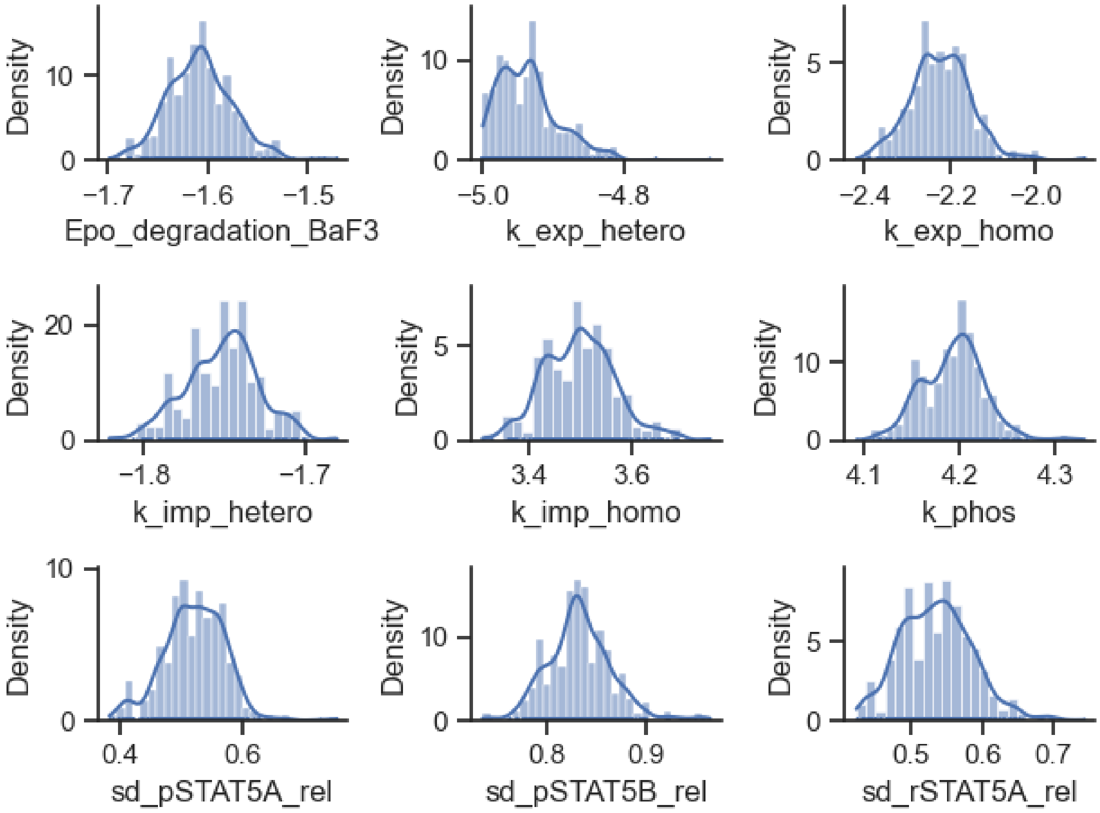

5. Storage

As the analysis itself is time consuming, it is neccesary to save the results for later usage. In this case it becomes even more apparent why one needs a **unified result object**, as otherwise saving will have to be adjusted each time one changes optimizer/sampler/profile startopint or other commonly changed things, or use an unsafe format such as pickling.

pyPESTO offers a unified result object and a very easy to use saving/loading-scheme:

[25]:
```
import tempfile

# create temporary file
fn = tempfile.mktemp(".h5")

# write result with write_result function.
# Choose which parts of the result object to save with
# corresponding booleans.
store.write_result(
    result=result_pypesto,
    filename=fn,
    problem=True,
```

```
    optimize=True,
    sample=True,
    profile=True,
)
```

[26]:
```
# Read result
result2 = store.read_result(fn, problem=True)
```

```
WARNING: You are loading a problem.
This problem is not to be used without a separately created objective.
```

[27]:
```
# plot profiles
visualize.sampling_1d_marginals(result2);
```

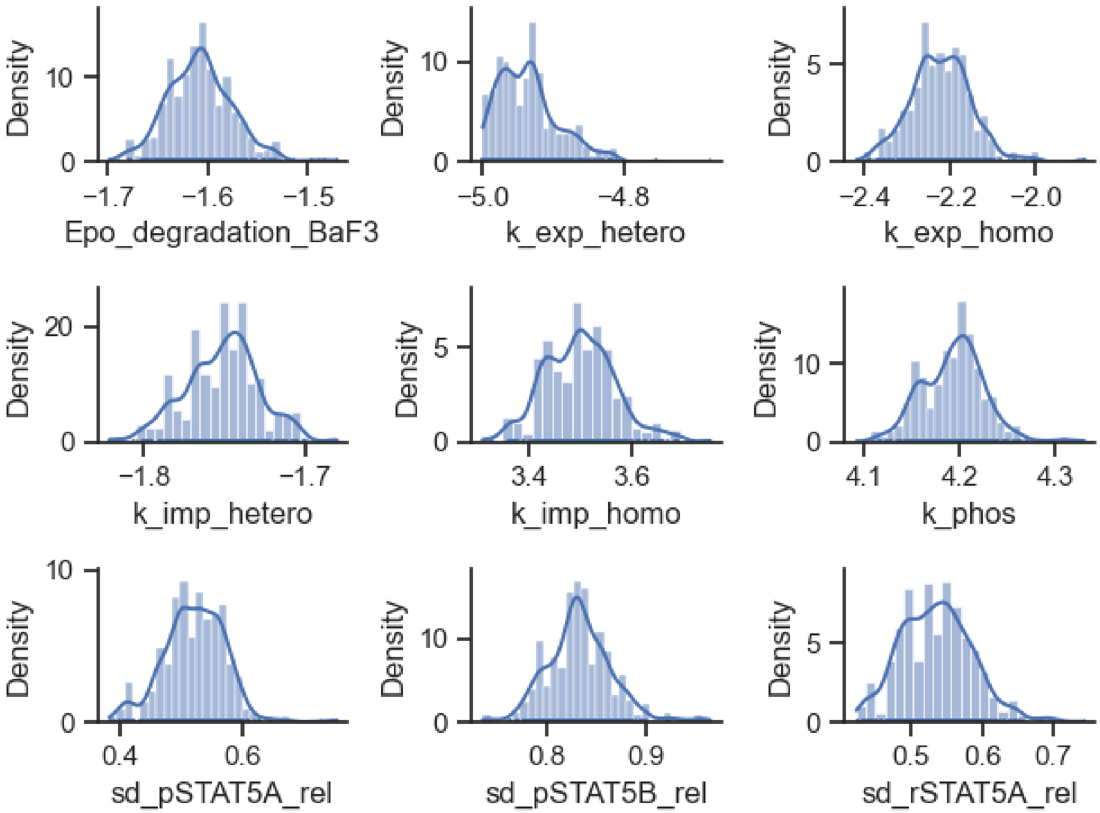

This concludes our brief rundown of a typical pyPESTO workflow and manual alternatives. In addition to what was shown here, pyPESTO provides a lot more functionality, including but not limited to visualization routines, diagnostics, model selection and hierarchical optimization. For further information, see the other example notebooks and the API documentation.

References

Balsa-Canto, Eva et al. (2016). "AMIGO2, a toolbox for dynamic modeling, optimization and control in systems biology". In: *Bioinformatics* 32.21, pp. 3357–3359.

Choi, Kiri et al. (Sept. 2018). "Tellurium: An extensible python-based modeling environment for systems and synthetic biology." In: *Bio Systems* 171, pp. 74–79. ISSN: 1872-8324. DOI: `10.1016/j.biosystems.2018.07.006`. ppublish.

Degasperi, Andrea, Dirk Fey, and Boris N Kholodenko (2017). "Performance of objective functions and optimisation procedures for parameter estimation in system biology models". In: *npj Syst Biol Appl* 3.1, p. 20. DOI: `10.1038/s41540-017-0023-2`.

Egea, J. A. et al. (2014). "MEIGO: An open-source software suite based on metaheuristics for global optimization in systems biology and bioinformatics". In: *BMC Bioinf.* 15.136. DOI: `10.1186/1471-2105-15-136`.

Fröhlich, Fabian et al. (Apr. 2021). "AMICI: high-performance sensitivity analysis for large ordinary differential equation models". In: *Bioinformatics* btab227. btab227. ISSN: 1367-4803. DOI: `10.1093/bioinformatics/btab227`. eprint: `https://academic.oup.com/bioinformatics/advance-article-pdf/doi/10.1093/bioinformatics/btab227/37371345/btab227.pdf`.

Hoops, S. et al. (2006). "COPASI – a COmplex PAthway SImulator". In: *Bioinformatics* 22.24, pp. 3067–3074. DOI: `10.1093/bioinformatics/btl485`.

Kaschek, Daniel et al. (2019). "Dynamic Modeling, Parameter Estimation, and Uncertainty Analysis in R". In: *J. Stat. Softw.* 88.10.

Mitra, Eshan D et al. (Sept. 2019). "PyBioNetFit and the Biological Property Specification Language." In: *iScience* 19, pp. 1012–1036. ISSN: 2589-0042. DOI: `10.1016/j.isci.2019.08.045`.

Raue, A. et al. (2015). "Data2Dynamics: a modeling environment tailored to parameter estimation in dynamical systems". In: *Bioinformatics* 31.21, pp. 3558–3560. DOI: `10.1093/bioinformatics/btv405`.

Schmiester, Leonard et al. (2020). "PEtab – interoperable specification of parameter estimation problems in systems biology". In: *arXiv preprint arXiv:2004.01154*.

Stapor, Paul et al. (2018). "PESTO: Parameter EStimation TOolbox". In: *Bioinformatics* 34.4, pp. 705–707. DOI: `10.1093/bioinformatics/btx676`.
